# Post renal transplant anemia: severity, causes and their association with graft and patient survival

**DOI:** 10.1186/s12882-019-1244-y

**Published:** 2019-02-13

**Authors:** Amir Schechter, Anat Gafter-Gvili, Daniel Shepshelovich, Ruth Rahamimov, Uzi Gafter, Eytan Mor, Avry Chagnac, Benaya Rozen-Zvi

**Affiliations:** 1 Medicine A, Rabin Medical Center, Beilinson Campus, Petah Tikva, Israel; 2 Department of Nephrology and Hypertension, Rabin Medical Center, Petah Tikva, Israel; 3 Department of Transplantation, Rabin Medical Center, Beilinson Campus, Petah Tikva, Israel; 4 Sackler Faculty of Medicine, Tel Aviv University, Tel Aviv, Israel

**Keywords:** Kidney transplantation, Anemia, Post-transplantation anemia, PTA

## Abstract

**Background:**

Post transplantation anemia (PTA) is common among kidney transplant patients. PTA is associated with increased graft loss and in most studies with increased mortality. However, the effect of the severity of anemia on this associations was not thoroughly evaluated.

**Methods:**

Patients who underwent kidney transplantation in Rabin Medical Center (RMC) were included in the study. Data were collected during the years 2002–2016. Anemia was defined as hemoglobin (Hb) level less than 12 g/dL in women and less than 13 g/dL in men, in accordance with World Health Organization (WHO) criteria. Severe anemia was defined as hemoglobin lower than 11 g/dL. Primary outcome was a composite of patient and graft survival. We used univariate and multivariate models to evaluate association between severity and specific causes of anemia with the outcomes. As the risk associated with anemia changed over time we analyzed the risk separately for the early and the late period (before and after 1251 days).

**Results:**

Our cohort included 1139 patients, 412 (36.2%) of which had PTA and 134 (11.7%) had severe anemia. On multivariable analysis, severe anemia was highly associated with the primary outcome at the early period (HR 6.26, 95% CI 3.74–10.5, *p* < 0.001). Anemia due to either AKI & acute rejection (11.9% of patients) or infection (16.7%), were associated with primary outcome at the early period (HR 9.32, 95% CI 5.3–26.41, *p* < 0.001 and HR 3.99, 95% CI 2.01–7.95, *p* < 0.001, respectively). There was non-significant trend for association between anemia due to Nutritional deficiencies (29.1%) and this outcome (HR 3.07, 95% CI 0.93–10.17, *p* = 0.067).

**Conclusion:**

PTA is associated with graft loss and mortality especially during the first three years. Anemia severity affects this association. An anemia workup is recommended for PTA.

**Electronic supplementary material:**

The online version of this article (10.1186/s12882-019-1244-y) contains supplementary material, which is available to authorized users.

## Background

Post-Transplant anemia (PTA) is common among kidney-transplant patients, as different studies report a prevalence of 20–51% at various time points after transplantation [1–6].

Early PTA is commonly defined as anemia during the first six months after the transplantation [5, 7]. It is usually due to iron deficiency [8, 9]. Early PTA is also influenced by the slowly increasing levels of the newly graft-produced-erythropoietin [8, 10–12].

Late PTA, seen in 23–36% of patients, is usually defined as anemia that occurs more than six months after transplantation, and can appear as late as up to eight years [5, 7, 13]. It is influenced by reduced allograft function, but also by iron deficiency, immunosuppressive medications, infections and other factors [2, 5, 14].

PTA has been shown to be negatively associated with long-term outcomes: higher rates of all-cause mortality [6, 15–18], graft failure [4, 19, 20], congestive heart failure [21] and a decline in estimated glomerular filtration rate (eGFR) [6, 13]. Surprisingly, severity level was not thoroughly studied, and only one of the above studies classified patients by hemoglobin levels [19]. Many factors have been associated with PTA including: iron deficiency, worsening renal function, recurrent transplantations, use of immunosuppressive medications, use of drugs acting on the renin angiotensin axis, infections, donor’s age and abnormally low levels of erythropoietin [11, 16, 22]. However, only a few studies showed an association between anemia etiology (mostly iron deficiency [23, 24]) with long-term prognosis.

Data are scarce regarding the association between anemia severity with patient and graft outcome, as well as the distribution of specific etiologies for anemia and their association with outcomes.

In this study, we investigated the association between anemia severity and etiology and long-term prognosis.

## Methods

### Study design and patients

Single center retrospective cohort study, using the Rabin Medical Center (RMC) kidney transplant registry. Inclusion criteria: all patients who underwent transplantation between the years 2002–2016, who had a functioning graft after six months, underwent a baseline anemia work-up at six months post-transplant, and a follow-up period of at least 12 months (i.e. 18 months from transplant). Exclusion criteria were age < 18 years, lack of documented clinic visits during the first 18 months and lack of a documented creatinine level after six months.

### Definitions

#### Anemia

Anemia was defined as hemoglobin (Hb) level less than 12 g/dL in women and less than 13 g/dL in men, in accordance with World Health Organization (WHO) criteria [25]. An episode of anemia was defined by at least two consecutives measurements of low Hb followed by two normal consecutive Hb values. Hb level was evaluated by routine complete blood count done at the Beilinson hospital laboratory using automated cell counter)Siemens, ADVIA 2120i).

#### Anemia severity

The definition of anemia severity varies between different studies. We chose to define severe anemia using the commonly used criteria of Hb < 11 g/dL [3, 18, 26, 27]. For evaluation of anemia severity we used the mean value of all hemoglobin levels documented during the anemic episode.

#### Causes of anemia

We classified anemia according to 5 main causes:

1. Acute rejection and/or acute kidney injury (AKI)
2. Infection (acute or chronic)
3. Nutritional deficiencies (iron, folic acid and/or vitamin B12)
4. Miscellaneous causes: hematological (hemoglobinopathy, hemolysis), neoplastic (lymphoma, plasma cell dyscrasia), bleeding (Gastro-intestinal, genitourinary or peri-procedural), hypothyroidism, chronic disease.
5. No obvious reason found: presumably due CKD and/or immunosuppressive drugs. We combined these causes since by definition all renal transplant recipients are considered CKD [28], and all of them receive immunosuppressive drugs.

#### Iron deficiency

Iron deficiency anemia was defined as anemia with a low ferritin and/or transferrin saturation level, based on CKD level, microcytosis and hypochromia, and according to KDOQI [29] (see Additional file 1 for detailed definitions).

#### Vitamin B12 deficiency

Total serum vitamin B12 is not a reliable biomarker of vitamin B12 availability [30, 31]. We chose to define B12 deficiency as < 300 pmol/L, as suggested by others for borderline and deficiency [32–35].

*Graft failure* was defined as chronic (≥3 months) treatment with hemodialysis, re-transplantation or death with functioning graft.

*Acute rejection* was defined by renal biopsy showing rejection of Banff score of 1A or higher.

*AKI* was defined, according to the KDIGO criteria [36].

#### Data collection

Patients’ characteristics were collected at baseline. For each patient, we documented all available episodes of anemia. Diagnosis of an episode of anemia was defined as the first time a decreased level of Hb was documented, according to the WHO criteria [25].

For each episode of anemia, full laboratory workup was collected. For patients without anemia, laboratory data were collected at six months following transplantation.

Acute infections based on culture, serological results and biopsy-proven acute graft rejections were collected from the electronic chart.

All possible causes of anemia for each episode were reviewed by two researchers (AS and BRZ) and in case of disagreement a third researcher evaluated the case (AG).

#### Outcomes

The primary outcome was the composite endpoint of graft failure (return to dialysis or re-transplantation) and all-cause mortality at the end of follow up.

Secondary outcomes were death censored graft failure (defined as re-establishment of long-term dialysis therapy, the need for re-transplantation) and all-cause mortality with a functioning graft.

#### Statistical analysis

Continuous data are presented as mean ± standard deviation or median and range, and dichotomous data as rate and percentages. Two-sample t-test and Mann–Whitney U-test were used for normally and non-normally distributed data, respectively. Differences in dichotomous variables were assessed by χ2 test. When numbers were small, the Fisher’s exact test was used instead of the χ2 test.

For the survival analysis we used a hierarchical method in order to attribute only one cause for anemia at a given time point. When anemia episode could be attributed to more than one cause and when two or more episodes of anemia due to a different cause occurred during the study period, the cause with higher hierarchy was considered as the anemia cause. Thus, every patient was assigned a single cause for his anemia (the highest-ranked cause in our model). The hierarchical order from lowest to highest was as follows: no identified cause, metabolic deficiencies, hemorrhage/hemolysis/hematologic causes, infection and AKI/rejection.

As not all anemia episodes occurred at six months we used a time dependent covariate model in which anemia was the time dependent covariate. Univariate and multivariate time varying Cox proportional hazard models were used, with the anemia incidence and severity as the time dependent covariate. The proportionality of the hazard was evaluated by adding the interaction for every covariate with time and assessing for the null hypothesis. Since anemia did not satisfy the propensity of the hazard assumption, we used a model with changing hazard. In order to find the point at which the hazard changed, we compared three possible models according to the quartiles of follow up time. We used the Wald statistics to evaluate the model with the best fit. The first quartile (180 to 1251 days) had the best fit according to the Wald statistics and was selected. When the proportionality of the hazard was evaluated for each time period (180 to 1251 days and 1251 days and onwards) the assumption was fully satisfied. As a result we analyzed the hazard separately for the early period (180 to 1251 days) and late period (1252 and onwards). As anemic patients were different in several factors we used extensive multivariate model to adjust for these differences.

The multivariate analysis was done using two models. First, we used a stepwise backwards regression model with *p*-value of 0.05 for inclusion and 0.15 for exclusion to adjust for the factors significantly associated with the outcome by univariate analysis (Model 1), and then we added to the model variables that might be associated with anemia or the outcome according to the literature (Model 2).

Covariates entered into the model included the following:

Recipient features including age, gender, kidney disease, pre-transplant diabetes mellitus (DM), pre-transplant heart disease, time on dialysis before transplantation, last Panel Reactive Antibodies (PRA) results classified as positive (10% or more) or negative, cytomegalovirus (CMV) serostatus, infection with hepatitis C (HCV) or hepatitis B virus (HBV) and former transplantations. Donor features included donor type (living or deceased), donor age, gender and HLA mismatch between the donor and recipient. Transplantation-related variables included cold ischemia time, induction therapy [none, anti thymocyte globulin (ATG), interleukin (IL)-2 antagonists and others], immunosuppression (tacrolimus, cyclosporine, mTOR inhibitor and antimetabolites and steroids alone), presence of delayed graft function, length of hospital stay (log transformed with the natural base in order to normalize data distribution), eGFR at 6, platelet count white blood cell (WBC), all at 6 months.

## Results

### Patients’ characteristics

During the study period 1404 adult patients underwent 1420 kidney transplantations. Of them, a total of 265 patients (18.9%) were excluded: 87 patients (6.2%) did not have functioning graft after 180 days, 86 patients (6.1%) had no follow up data, 77 patients (5.5%) had missing baseline data, 15 patients (1.1%) had other organ transplantation (heart, lung, liver and pancreas). Anemic episodes after second transplantation during the study period were excluded since those 16 patients were considered to achieve the study outcome. Therefore, 1139 patients with functioning graft were available for analysis, Additional file 1: Figure S1. Of them, 412 patients (36.2%) had at least one episode of anemia within 6–18 months after transplantation, with a total of 500 events. Distribution of anemia episodes is as follows: 341 (82.8%) had one episode, 59 (14.3%) had two separate episodes, 11 (2.7%) had three separate episodes, and two (0.5%) had 4 episodes.

Table 1 depicts the demographic, baseline and medication characteristics, according to patients who developed anemia compared to those who did not, and according to severity of anemia. Anemic patients were more likely to have diabetes and cardiovascular comorbidities and had an older donor. Approximately 95% of all patients were treated with a similar immunosuppressive regimen consisting of a calcineurin inhibitor and mycophenolic acid. Distribution of the causes of anemia is presented in Table 2. Nutritional deficiencies were the most common. Of them, the most common deficiency was iron deficiency, which was diagnosed in 143 patients (34.7% of anemic patients) and in 157 separate events (31.4%).

**Table 1:** Demographic and baseline characteristics of the anemic and non-anemic patients

	Anemia			No anemia (*n* = 727)	Total (*n* = 1139)
	Mild (*n* = 278)	Severe (*n* = 134)	All (*n* = 412)		
Mean age at transplant (mean ± SD)	50.7 ± 15.8	45.9 ± 16.3	49.1 ± 16.1	47.5 ± 14.1	48.1 ± 14.9
Male sex *n* (%)	60 (21.6)	81 (60.4)	141 (34.2)	236 (32.5)	377 (33.1%)
BMI (Kg/m^2)	26.7 ± 5	24.7 ± 6	26.1 ± 5.4	26 ± 4.8	26 ± 5
HCV positive *n* (%)	3 (1.1)	3 (2.4)	6 (1.6)	33 (4.5)	39 (3.5)
HBV positive *n* (%)	4 (1.5)	4 (3.2)	8 (2.1)	14 (1.9)	22 (2)
Donor CMV positive n (%)	202 (77.1)	93 (74.4)	295 (76.2)	592 (81.4)	887 (79.6)
Recipient CMV positive *n* (%)	220 (84)	111 (88.8)	331 (85.5)	647 (89)	978 (87.8)
Original renal disease					
Glomerulonephritis	64 (20.9)	42 (28.2)	106 (23.3)	227 (29.9)	333 (26.6)
CAKUT	37 (12.1)	22 (17.5)	59 (13)	67 (8.4)	126 (10.1)
Diabetic nephropathy	72 (23.5)	36 (24.2)	108 (23.7)	132 (16.6)	240 (19.2)
Polycystic kidney disease (PKD)	29 (9.5)	12 (8.1)	41 (9)	102 (12.8)	143 (11.4)
Genetic disease (excluding PKD)	11 (3.6)	4 (2.7)	15 (3.3)	18 (2.3)	33 (2.6)
Chronic interstitial nephritis	8 (2.6)	4 (2.7)	12 (2.6)	13 (1.6)	25 (2)
No diagnosis/unknown	85 (27.8)	29 (19.5)	114 (25.1)	238 (29.9)	352 (28.1)
Length of hospitalization (ln, days)	2.4 ± 0.5	2.5 ± 0.6	2.4 ± 0.5	2.5 ± 0.5	2.4 ± 0.5
Mean duration of dialysis (months)	32.8 ± 37.3	36.8 ± 45.7	34.1 ± 40.2	34.3 ± 38.4	34.2 ± 39
Previous renal transplantation *n* (%)					
0	255 (91.7)	110 (82.1)	365 (88.6)	643 (88.4)	1008 (88.5)
≥1	23 (8.3)	24 (17.9)	47 (11.4)	84 (11.6)	131 (11.5)
Living donor (%)	167 (60.1)	74 (55.2)	241 (58.5)	427 (58.7)	668 (58.6)
Mean donor age (years)	48.9 ± 14.3	47 ± 14.6	48.3 ± 14.4	42.66 ± 13.9	44.7 ± 14.3
HLA Mismatch (mean ± SD)	4.2 ± 1.6	4.2 ± 1.5	4.2 ± 1.6	4 ± 1.6	4.1 ± 1.6
Mean cold ischemia time (hours)	5:06 ± 6:31	5:09 ± 7:08	5:07 ± 6:42	6:16 ± 7.56	5:52 ± 7.33
PRA > 10% (%)	31 (11.2)	22 (16.4)	53 (12.9)	79 (10.9)	132 (11.6)
Delayed graft function *n* (%)	48 (17.3)	31 (23.1)	79 (19.2)	124 (17.1)	203 (17.8)
Co-morbidities *n* (%)					
Diabetes	96 (34.5)	44 (32.8)	140 (34)	166 (22.8)	306 (26.9)
Hypertension	218 (86.2)	100 (82.6)	318 (85)	610 (87.3)	928 (86.5)
Cardiovascular	76 (27.3)	30 (22.4)	106 (25.7)	123 (16.9)	229 (20.1)
Laboratory workup at 6 months:					
eGFR (ml/min/1.73 m^2)	55.8 ± 19.6	52.3 ± 22.7	54.7 ± 20.7	63.3 ± 19.5	60.2 ± 20.3
Mean Hemoglobin (g/dL)	12.1 ± 1.1	10.15 ± 1.5	11.7 ± 1.4	13.4 ± 1.7	12.8 ± 1.8
Hematocrit (%)	37.1 ± 3.6	33.9 ± 4.7	36.1 ± 4.3	41.1 ± 5.2	39.2 ± 5.4
MCV (fL)	87.7 ± 6.4	87.2 ± 7.9	87.5 ± 6.9	88.3 ± 5.8	88 ± 6.3
RDW (%)	14.5 ± 1.5	14.7 ± 1.5	14.6 ± 1.5	14.3 ± 1.2	14.4 ± 1.3
Platelets (X10^3/microL)	196.5 ± 68.1	229.8 ± 90.5	207.3 ± 77.5	208.7 ± 65.4	208.2 ± 70.1
Leukocytes (X10^3/microL)	6.4 ± 2.2	6.7 ± 3.4	6.5 ± 2.6	7.4 ± 2.6	7 ± 2.7
Iron (microgram/dL)	71.2 ± 34.4	63.7 ± 35.9	68.1 ± 35.2	77.6 ± 36.9	72.9 ± 36.4
Transferrin (mg/dL)	219.2 ± 42.6	197.8 ± 59.1	210.3 ± 51.1	221.3 ± 49.9	215.8 ± 50.8
Transferrin saturation (%)	23.6 ± 13	24.8 ± 15.7	24.1 ± 14.2	25.7 ± 14.6	24.9 ± 14.4
Ferritin (ng/mL)	370.2 ± 393.2	461.4 ± 537	408.6 ± 460.1	388.8 ± 458.5	399.5 ± 458.8
Albumin *n* (%)	4.04 ± 0.5	4.02 ± 0.5	4.04 ± 0.5	4.15 ± 0.39	4.1 ± 0.45
Folic acid (nmol/L)	15.3 ± 9.7	15.9 ± 12.1	15.5 ± 10.6	16.7 ± 9.9	16.1 ± 10.2
Vitamin B12 (pmol/L)	474.7 ± 299.9	487.9 ± 284.1	479 ± 294.1	413.9 ± 213	16.1 ± 10.2
FK506 level (ng/ml)	8.4 ± 2.6	8.4 ± 3	8.4 ± 2.8	8.8 ± 3	8.7 ± 2.9
Immunosuppressive drugs					
Tacrolimus	245 (93.5)	110 (88)	355 (91.7)	661 (90.9)	1016 (91.2)
Cyclosporine	8 (3.1)	9 (7.2)	17 (4.4)	31 (4.3)	48 (4.3)
mTOR inhibitors	6 (2.3)	4 (3.2)	10 (2.6)	22 (3)	32 (2.9)
Other	3 (1.1)	2 (1.6)	5 (1.3)	13 (1.8)	18 (1.6)
Erythropoietin treatment (%)	7 (2.5)	8 (5.9)	15 (3.6)	0 (0)	15 (1.3)

Abbreviations: *BMI* Body mass index, *HCV* Hepatitis C virus, *HBV* Hepatitis B virus, *CMV* Cytomegalovirus, *CAKUT* Congenital anomalies of the kidney and urinary tract, HLA, human leukocyte antigen, *PRA* Panel reactive antibody eGFR, Estimated glomerular filtration rate, *MCV* mean corpuscular volume, *RDW* Red blood cell distribution width, *mTOR* mammalian target of

**Table 2:** Causes of anemia

Causes	Patients *n* (% of anemic patients)	Events *n* (% of anemia events)
Nutritional deficiencies		
Iron deficiency	143 (34.7)	157 (31.4)
Vitamin B12	98 (23.8)	105 (21)
Folic acid	41 (9.9)	43 (8.6)
Acute rejection	17 (4.1)	22 (4.4)
Acute kidney injury	47 (11.4)	48 (9.6)
Infection		
Urinary tract infection	32 (7.8)	33 (6.6)
Pulmonary^a^	11 (2.7)	11 (2.2)
Skin^b^	11 (2.7)	11 (2.2)
Miscellaneous^c^	11 (2.7)	11 (2.2)
BK Virus	26 (6.3)	31 (6.2)
CMV viremia	16 (3.9)	16 (3.2)
Herpes zoster	4 (1)	4 (0.8)
B19 Parvovirus	1 (0.2)	1 (0.2)
Hematological	12 (2.9)	15 (3)
Thalassemia trait	5 (1.2)	6 (1.2)
PTLD	2 (0.5)	3 (0.6)
Miscellaneous^d^	5 (1.2)	6 (1.2)
Bleeding	39 (9.5)	39 (7.8)
Peri-procedural	20 (4.8)	20 (4)
Monthly menstruation	11 (2.7)	11 (2.2)
Miscellaneous^e^	8 (1.9)	8 (1.6)

^a^Pneumonia, viral pneumonitis, upper respiratory tract infection

^b^Cellulitis, diabetic foot, herpes zoster

^c^Gastroenteritis (bacterial and viral, including CDT infection), osteomyelitis, arthritis, endocarditis, meningitis, sinusitis, cholangitis, intra-abdominal abscess, fungal cutaneous infection and fever of unknown origin

^d^Myelodysplastic syndrome, plasma cell disease (multiple myeloma), myeloproliferative disorder (myelofibrosis), myelophthisis (renal cell carcinoma metastasized to bone marrow) and exacerbation of a known ITP with an effect on the erythrocytic lineage

^e^Hemoptysis, traumatic bleeding after a fall, retro-peritoneal bleeding, macro-hematuria, GI bleeding

Abbreviations: *CMV* Cytomegalovirus, *PTLD* Post-transplant lymphoproliferative disorder

### Outcomes

During a median follow up of 5.5 years (interquartile range 3.4–8.7 years), a total of 265 deaths or graft loss occurred (23.3% of the entire cohort). One hundred seventy two patients died (15.1%) and graft loss occurred in 129 patients (11.3%).

The effect of anemia, anemia severity and specific causes on the outcomes are presented for the primary composite outcome (Table 3), death-censored graft survival (Table 4), and all-cause mortality (Table 5).

**Table 3:** Graft failure and all-cause mortality

		Univariate			Multivariate		
		HR	95% CI	*P* value	HR	95% CI	*P* value
Presence of anemia	Early	3.64	2.34–5.66	< 0.001	2.94	1.85–4.66	< 0.001
	Late	1.57	1.09–2.26	0.015	1.44	0.97–2.13	0.074
Severity of anemia							
Mild (Hb > 11 g/dL)	Early	1.89	1.07–3.31	0.027	1.42	0.79–2.54	0.244
	Late	1.5	0.99–2.27	0.056	1.15	0.6–2.17	0.327
Severe (Hb < 11 g/dL)	Early	8.01	4.92–13.04	< 0.001	6.26	3.74–10.5	< 0.001
	Late	1.79	0.98–3.25	0.058	1.65	0.88–3.11	0.121
Causes of anemia							
Acute rejection and/or acute kidney injury	Early	12.3	7.2–21.0	< 0.001	9.32	5.3–16.41	< 0.001
	Late	2.32	1.13–4.75	0.022	2.06	0.97–4.39	0.061
Infection	Early	5.02	2.58–9.78	< 0.001	3.99	2.01–7.95	< 0.001
	Late	1.42	0.66–3.06	0.365	1.26	0.57–2.79	0.564
Nutritional deficiencies	Early	3.12	1.10–8.85	0.032	3.07	0.93–10.17	0.067
	Late	0.67	0.09–4.74	0.694	1.04	0.142–7.56	0.971
Miscellaneous	Early	2.43	1.22–4.83	0.012	1.82	0.85–3.48	0.133
	Late	1.75	0.96–3.2	0.068	1.44	0.76–2.7	0.263
No reason found	Early	0.65	0.197–2.11	0.467	0.59	0.18–1.94	0.383
	Late	1.38	0.77–2.47	0.281	1.29	0.7–2.37	0.415

Abbreviations: *HR* Hazard ratio, *CI* Confidence interval

Covariates included in the multivariate models are specified in the supplementary tables

**Table 4:** Death censored graft failure

		Univariate			Multivariate		
		HR	95% CI	P value	HR	95% CI	*P* value
Presence of anemia	Early	4.06	2.17–7.57	< 0.001	3.15	1.63–6.08	0.001
	Late	2.17	1.33–3.55	0.002	1.853	1.09–3.16	0.024
Severity of anemia							
Mild (Hb > 11 g/dL)	Early	1.76	0.77–4.03	0.179	1.35	0.56–3.25	0.5
	Late	2	1.14–3.5	0.015	1.66	0.91–3.01	0.096
Severe (Hb < 11 g/dL)	Early	9.78	5.0–19.13	< 0.001	7.6	3.69–15.65	< 0.001
	Late	2.65	1.25–5.64	0.011	2.61	1.15–5.92	0.021
Causes of anemia							
Acute rejection and/or acute kidney injury	Early	18.18	9.15–32.32	< 0.001	12.94	6.14–27.28	< 0.001
	Late	3.93	1.68–9.29	0.002	4.14	1.64–10.45	0.003
Infection	Early	3.47	1.15–10.47	0.027	3.01	0.97–9.33	0.056
	Late	1.36	0.42–4.39	0.603	1.09	0.33–3.64	0.885
Nutritional deficiencies	Early	3.25	0.74–14.14	0.117	1.63	0.21–12.51	0.636
	Late	1.47	0.2–10.75	0.703	1.88	0.25–14.13	0.538
Miscellaneous	Early	2.75	1.07–7.1	0.036	2.14	0.81–5.65	0.125
	Late	2.57	1.2–5.49	0.015	1.91	0.86–4.23	0.11
No reason found	Early^a^						
	Late	1.75	0.79-3.88	0.17	1.58	0.69–3.64	0.28

Abbreviations: *HR* Hazard ratio, *CI* Confidence interval

Covariates included in the multivariate models are specified in the supplementary tables

^a^No outcome events

**Table 5:** All-cause mortality

		Univariate			Multivariate		
		HR	95% CI	*P* value	HR	95% CI	*P* value
Presence of anemia	Early	3.24	1.73–6.08	< 0.001	2.27	1.17–4.4	0.015
	Late	1.1	0.63–1.92	0.735	0.8	0.44–1.47	0.469
Severity of anemia							
Mild (Hb > 11 g/dL)	Early	1.11	0.93–4.31	0.077	1.49	0.67–3.32	0.327
	Late	1.11	0.59–2.07	0.75	0.94	0.48–1.86	0.862
Severe (Hb < 11 g/dL)	Early	6.36	3.1–13.03	< 0.001	5.42	2.51–11.68	< 0.001
	Late	1.09	0.39–2.99	0.871	0.97	0.32–2.93	0.962
Causes of anemia							
Acute rejection and/or acute kidney injury	Early	6.88	2.83–16.76	< 0.001	4.49	1.77–11.34	0.002
	Late	1.04	0.26–4.26	0.954	0.53	0.12–2.31	0.41
Infection	Early	6.45	2.76–15.91	< 0.001	4.27	1.77–10.25	0.001
	Late	1.48	0.54–4.08	0.444	1.16	0.40–3.36	0.779
Nutritional deficiencies	Early	2.99	0.69–13.05	0.143	4.03	0.9–17.99	0.068
	Late^a^						
Miscellaneous	Early	2.12	0.78–5.79	0.142	1.23	0.44–3.47	0.693
	Late	1.08	0.39–2.99	0.88	0.89	0.3–2.61	0.834
No reason found	Early	1.24	0.36–4.26	0.731	0.91	0.26–3.21	0.879
	Late	1.1	0.47–2.6	0.828	0.86	0.35–2.08	0.733

Abbreviations: *HR* Hazard ratio, *CI* Confidence interval

Covariates included in the multivariate models are specified in the supplementary tables

^a^No outcome events

#### I. Effect of anemia on outcomes

In a univariate analysis, anemia was significantly associated with graft loss or mortality in the early period [hazard ratio (HR) 3.64, 95% confidence interval (CI) 2.34–5.66, *p* < 0.001] while at the late period the association was weaker (HR 1.44, 95% CI 0.97–2.13, 0.07), Table 3.

Multivariate analysis showed the same significant association for the early period (HR 2.94, 95% CI 1.86–4.66, *p* < 0.001, model 2), and a nonsignificant association for the later period (HR 1.44, 95% CI 0.97–2.13, 0.074).

Similar results were obtained for the outcome of all-cause mortality during the early period: (HR 3.24, 95% CI 1.73–6.08, < 0.001) and (HR 2.27, 95% CI 1.17–4.4, *p* = 0.015, model 2) for univariate and multivariate analyses, respectively. For the late period there was no association between anemia and mortality for both univariate and multivariate (Table 4).

Anemia was significantly associated with death censored graft loss during the early period by both univariate (HR 4.06, 95% CI 2.17–7.57, *p* < 0.001) and multivariate analysis (HR 3.15, 95% CI 1.63–6.08, *p* = 0.001, model 2). Anemia was also associated with death censored graft loss at the late period only with smaller hazard (HR 2.17, 95% CI 1.33–3.55, *p* = 0.002) and (HR 1.853, 95% CI 1.09–3.16, *p* = 0.024, model 2) for univariate and multivariate analysis respectively (Table 5).

#### II. Effect of Anemia severity on outcomes

In a univariate analysis of the three groups according to anemia severity (no anemia, mild anemia and severe anemia), all anemia groups were significantly associated with graft loss or mortality during the early period. However, the HR in the severe anemia group was higher than in the mild anemia group (HR 1.89, 95% CI 1.07–3.31, *p* = 0.027, for mild anemia and 8.01, 95% CI 4.92–13.04, *p* < 0.001, for severe anemia). By a multivariate analysis, severe anemia was still strongly associated with this endpoint at the early period (HR 6.26, 95% CI 3.74–10.5, *p* < 0.001, model 2). However, for mild anemia there was no significant association (HR 1. 1.42, 95% CI 0.79–2.54, *p* = 0.244, model 2). Both mild and severe anemia were not significantly associated with mortality and graft loss during the late period (Table 3).

In contrast, only severe anemia was associated with all-cause mortality at the early period (HR 6.36, 95% CI 3.1–13.03, *p* < 0.001), while for mild anemia the association was not significant (HR 2, 95% CI 0.93–4.31, *p* = 0.077). The association between severe anemia and mortality remained significant in the multivariate analysis (HR 5.42, 95% CI 2.51–11.68, *p* < 0.001, model 2). There was no association between mild and severe anemia and mortality at the late period (Table 4).

When death censored graft survival was evaluated, severe anemia was significantly associated with this outcome, at both the early and late periods (HR 9.78, 95% CI 5.0–19.13, *p* < 0.001, and HR 2.65, 95% CI 1.25–5.64, *p* = 0.011, for early and late period respectively). In a multivariate analysis, the results were not changed: early period (HR 7.6, 95% CI 3.69–15.65 *p* < 0.001, model 2), late period (HR 2.61, 95% CI 1.15–5.92, *p* = 0.021, model 2). Mild anemia was not associated with death censored graft survival during the early period by both univariate and multi variate analysis (Table 5). In contrast, there was an association between mild anemia and death censored graft survival during the late period but, this association became non-significant by multivariate analysis (HR 2, 95% CI 1.14–3.5, *p* = 0.015, for univariate and 1.66, 95% CI 0.91–3.01, *p* = 0.096, for multivariate).

#### III. Effect of specific causes of anemia on outcomes

In a univariate analysis, AKI & acute rejection (HR 12.3, 95% CI 7.2–21.0, *p* < 0.001), infections (HR 5.02, 95% CI 2.58–9.78, *p* < 0.001), nutritional deficiencies (HR 3.12, 95% CI 1.10–8.85, *p* = 0.032) and miscellaneous reasons (HR 2.43, 95% CI 1.22–4.83, *p* = 0.012) were all associated with graft loss or mortality during the early period. In contrast, anemia of unidentified cause was not significantly associated with graft loss or mortality. In a multivariate analysis the results were similar to those found in the univariate analysis for anemia due to AKI & acute rejection as well as infections. For anemia due to nutritional deficiencies (HR 3.07, 95% CI 0.93–10.17, *p* = 0.067) and miscellaneous reasons (HR 1.82, 95% CI 0.85–3.48, *p* = 0.133) the association was non-significant.

When the late period was evaluated by univariate analysis only AKI & acute rejection (HR 2.32, 95% CI 1.13–4.75, *p* = 0.022) was associated with graft loss and mortality. By multivariate analysis the association with graft loss and mortality at the late period became a non-significant trend (HR 2.06, 95% CI 0.97–4.39, *p* = 0.061).

When all-cause mortality during the early period was evaluated, only anemia due to AKI and/or acute rejection (HR 6.88, 95% CI 2.83–16.76, *p* < 0.001 and HR 4.49, 95% CI 1.77–11.34, *p* = 0.002, for univariate and model 2 respectively) and infection (HR 6.45, 95% CI 2.76–15.91, *p* < 0.001, HR 4.27, 95% CI 1.77–10.25, *p* = 0.001, for univariate and model 2 respectively) were associated with increased mortality. In contrast no association was found between any etiology for anemia and mortality at the late period.

Death censored graft loss at the early period was highly associated with AKI & acute rejection (HR 18.18, 95% CI 9.15–32.32, *p* < 0.001, and HR 12.94, 95% CI 6.14–27.28, *p* < 0.001, for univariate and model 2 respectively). Anemia due to infections was significantly associated with this outcome only by univariate analysis (HR 3.47, 95% CI 1.15–10.47, *p* = 0.002, and HR 3.01, 95% CI 0.97–9.33, *p* = 0.056, for univariate and model 2 respectively) with similar results for anemia due to miscellaneous reasons (HR 2.75, 95% CI 1.07–7.1, *p* = 0.037, and HR 2.14, 95% CI 0.81–5.65, *p* = 0.125, for univariate and model 2 respectively).

Death censored graft loss at the late period was associated only with AKI & acute rejection and the association remained significant after multivariate adjustment (HR 3.93, 95% CI 1.68–9.29, *p* = 0.002, and HR 4.14, 95% CI 6.14–27.28, *p* = 0.003, for univariate and model 2 respectively). Anemia due to miscellaneous causes was associated with death censored graft loss only by univariate analysis and the association was not significant after multivariate adjustment (HR 2.57, 95% CI 1.2–5.49, *p* = 0.015, and HR 1.91, 95% CI 0.86–4.23, *p* = 0.110, for univariate and model 2 respectively).

Both infection and rejection, known causes for anemia, are greatly impacted by immunosuppression. Since immunosuppression may affect the outcomes, we conducted another analysis for the primary outcome including only the 877 patients treated with tacrolimus (with at least 3 available tacrolimus levels during the first six months). We included the mean tacrolimus level as a covariate, as can be seen in Additional file 1: Tables S1-S3. The results were not very different.

The full multivariable model with all of the covariables is shown in Additional file 1: Tables S4-S12.

## Discussion

In this cohort study of 1139 kidney transplant recipients we demonstrated that the severity of anemia is strongly associated with graft failure and mortality. Furthermore, we identified various causes of late PTA, which were associated with prognosis.

PTA has previously been shown to be associated with the composite outcome of all-cause mortality and graft loss [6, 17, 18, 27, 37–39], as well as the separate components: death-censored graft survival [4, 19, 20] and all-cause mortality [6, 15–18]. However, the association between PTA and increased mortality is inconsistent, as several studies failed to show association with all-cause mortality, despite an association with graft loss [4, 19, 40–43]. Our study shows an association between anemia and the primary outcome of graft failure and mortality, as well as with mortality alone.

In our study, anemia was diagnosed in 36% of the cohort, in agreement with previous studies which showed a late PTA prevalence of 20–40% [1–6, 23, 27, 37, 38, 42, 44].

All-cause mortality was significantly associated in our study only with severe anemia. These results are in accordance with the findings by Heinze et al. [15] who found a negative correlation between mortality and hemoglobin level in patients with late PTA. The results of our study may explain the results of the studies that showed no association of late PTA with mortality [4, 19, 40–43]. Most patients in these studies had mild anemia with mean hemoglobin above 11 g/dl. Death censored graft loss was significantly associated with late PTA by univariate and multivariate analyses in both anemic groups. However, this association was weaker in the mild anemia group. Huang et al. [19] showed that only the more severe levels of anemia (defined as hemoglobin < 11 g/L and < 10 g/L for men and women, respectively) were associated with lower 3 and 5-year graft survival rates, but did not find an association with all-cause mortality.

Many studies described various causes for PTA. However, most studies did not address the importance of diagnosing the exact etiology for anemia. Nevertheless, two studies showed that iron deficiency [23] and the percentage of hypochromic RBC’s [24] are predictors of high rates of all-cause mortality. To the best of our knowledge, our study is the first to thoroughly examine the association between specific etiologies of anemia and prognosis. We demonstrated that the specific etiologies of anemia are associated with prognosis: anemia due to AKI, acute rejection, infection or nutritional deficiencies is associated with higher risk of death or graft loss, while anemia in which no specific cause was found is not.

Regarding the frequency of the specific etiologies, iron deficiency anemia was diagnosed in 35% of anemic patients of our population (13% of the cohort), in accordance with previous reports [2, 23].

Folic acid deficiency was diagnosed in 10% of anemic patients, a relatively low rate as compared to 23–41% in other studies [2, 45, 46]. This may be due to the widespread folic acid supplementation of food to prevent neural birth defects.

Vitamin B12 was diagnosed in 24% of anemic patients, in accordance with the 17–24% range reported in other studies [34, 45, 46].

Our study has several strengths: first, our cohort is large, with over 1000 transplant recipients, and about 400 patients with anemia. In addition, there is an adequate follow up time of over 5.5 years, with separate analyses of the first 3.4 years and a later period. Second, in contrast to other studies, our study was not a cross-sectional, and the presence of anemia was evaluated over a time span of 12 months, and incorporated clinical data derived from routine clinic visits, hospitalizations, and laboratory workup. Anemia severity was evaluated by using the mean Hb value for the whole episode which is probably better than using an arbitrary single value. Furthermore, since we conducted separate analyses for two time periods after transplant, an “early” period of about 3.4 years (1251 days) and a later period, we could show that the association between anemia at any time point between 6 and 18 months after transplantation and graft failure and mortality was seen only during the earlier period. We show for the first time that the risk for mortality associated with anemia changes over time. Although the study is retrospective, and one cannot assume causality between anemia and outcomes, this temporal association in time may elucidate the possible effect that anemia has on outcomes.

Several limitations merit consideration. First, due to the retrospective design of the study, we can only show an association between severity of anemia and outcomes, rather than causality. Some anemia events had multiple causes. Although we used a hierarchical strategy in which every event was assigned a single cause, the effect of each cause cannot be evaluated. In addition, our study had a relatively large group of patients with anemia that we considered as an unknown cause. It is most likely that these patients had anemia due to chronic renal failure (as the KDIGO recommendations define all patients post transplant as CKD patients [28, 47]) and/or due to immunosuppressive therapy. Although CKD (eGFR< 60 ml/min/1.73 m2, CKD-3-5) is an established cause for anemia, we chose not to include renal function as a separate cause for anemia, as reduced renal function is a well-known risk factor for graft loss. However, eGFR was included in our multivariate models so any effect of renal function on graft survival was adjusted for. Immunosuppressive drugs are another established cause of anemia [48–50] that was not included in our study, as the vast majority of our cohort (≈95%) was treated by the same protocol of calcineurin inhibitor with mycophenolic acid, the effect of medication could not be properly evaluated in this population. Notably, since this is a retrospective study, there were a few differences between the anemic and non-anemic groups, mainly, anemic patients had an older donor, diabetes and cardiovascular comorbidity. Nevertheless, anemia was indeed shown to be an independent factor for graft failure and death. Another limitation is the lack of patients of African American, Hispanic or Asian origin in our population, so generalization of our results to these population should be done with caution.

Our findings should encourage physician to thoroughly evaluate patients with anemia. All patients with severe anemia are at risk for a graft failure and mortality, and even for mild anemia, rejection and infections should be excluded and patients should be evaluated for metabolic deficiencies and treated accordingly.

Our study shows an association between severe anemia and outcomes. Therefore, although not assessed in this study, it seems reasonable that an attempt should be made to treat anemia. The management of PTA usually starts with diagnosing and treating all reversible underlying causes. However, there is no specific recommendation for treatment of kidney transplant recipients in KDIGO guidelines [36]. Several issues regarding specific treatment are unclear. Most importantly, the target Hb level for treatment. Second, the type of treatment, erythropoiesis stimulating agents (ESA) and/or iron.

Data on treatment of late PTA are limited, but show a beneficial effect for raising the Hb level. These studies tried to assess the optimal Hb level with ESA administration. A randomized trial by Choukroun et al. [13] demonstrated that higher Hb level was associated with graft survival. Normalizing PTA (to a Hb level of 13–15 g/dl) with epoetin beta compared with partial correction of Hb to 10.5 to 11.5 g/dL, was associated with reduction in the rate of decline of eGFR and progression to ESRD, and improved death-censored graft survival [13]. Heinze et al. [15], in a cross sectional retrospective cohort study found that anemia (Hb < 12.5 g/dl) was significantly associated with mortality, in both patients with or without (ESA). In patients without ESA, a spontaneous rise in Hb was associated with decreased mortality at any level. In patients treated with ESA, improvement of anemia up to 12.5 g/dL was associated with decreased mortality as well. Further increments in Hb level led to a tendency of increased rate of mortality, which became significant at Hb level above 14 g/dL [15].

In contrast to the abundance of data from RCTs demonstrating the efficacy of iron in the CKD population, data regarding treatment with iron in PTA are scarce. Our group reported in a retrospective cohort study of 81 patients that intravenous iron administration after transplantation increases hemoglobin level and delays eGFR decline, both of which were more pronounced in lower hemoglobin levels (mean 9.4 ± 1.2 g/dL) [51]. Yet, a small RCT of 104 patients comparing oral with a single dose of intravenous iron after transplantation did not show a difference in time to anemia correction [52]. However, this trial was conducted in the post-operative period, and does not address late PTA.

Based on our findings of improved prognosis with a lesser degree of anemia and the finding in literature of response to treatment [13, 15], it might be prudent to raise the Hb level. However, the optimal correction level and the best treatment modality are unclear.

## Conclusions

In conclusion, we found an association between anemia and mortality and graft failure which is related to the severity and causes of anemia. Anemia workup is highly warranted in order to find the specific underlying causes. Future research should focus on the target Hb level, and the appropriate use of ESA and iron.

## Additional file

Additional file 1: Iron deficiency definitions. **Figure S1.** Flow of patients. **Table S1.** Composite outcome, with tacrolimus level. **Table S2.** Death censored graft failure, with tacrolimus level. **Table S3.** All-cause mortality, with tacrolimus level. **Table S4.** Composite outcome, presence of anemia, without tacrolimus level. **Table S5.** Composite outcome, severity of anemia, without tacrolimus level. **Table S6.** Composite outcome, causes of anemia, without tacrolimus level. **Table S7.** Death censored graft failure, presence of anemia, without tacrolimus level. **Table S8.** Death censored graft failure, Severity of anemia, without tacrolimus level. **Table S9.** Death censored graft failure, causes of anemia, without tacrolimus level. **Table S10.** All-cause mortality, presence of anemia, without tacrolimus level. **Table S11.** All-cause mortality, severity of anemia, without tacrolimus level. **Table S12.** All-cause mortality, causes of anemia, without tacrolimus level. (DOCX 59 kb)

## Abbreviations

AKI: Acute kidney injury
CI: Confidence interval
CKD: Chronic kidney disease
CKD-EPI: Chronic Kidney Disease Epidemiology Collaboration
eGFR: Estimated glomerular filtration rate
ESRD: End stage renal disease
Hb: Hemoglobin
HR: Hazard ratio
KDIGO: Kidney Disease: Improving Global Outcomes
MCV: Mean corpuscular volume
PTA: Post transplantation anemia
RCT: Randomized controlled trial
WHO: World Health Organization
