# Supplementary material for: Post renal transplant anemia: severity, causes and their association with graft and patient survival
Source: BMC Nephrol. 2019 Feb 13;20:51. doi: 10.1186/s12882-019-1244-y (PMC6374899; doi:10.1186/s12882-019-1244-y)
Supplement: Supplementary file 1 — Iron deficiency definitions. Figure S1. Flow of patients. Table S1. Composite outcome, with tacrolimus level. Table S2. Death censored graft failure, with tacrolimus level. Table S3. All-cause mortality, with tacrolimus level. Table S4. Composite outcome, presence of anemia, without tacrolimus level. Table S5. Composite outcome, severity of anemia, without tacrolimus level. Table S6. Composite outcome, causes of anemia, without tacrolimus level. Table S7. Death censored graft failure, presence of anemia, without tacrolimus level. Table S8. Death censored graft failure, Severity of anemia, without tacrolimus level. Table S9. Death censored graft failure, causes of anemia, without tacrolimus level. Table S10. All-cause mortality, presence of anemia, without tacrolimus level. Table S11. All-cause mortality, severity of anemia, without tacrolimus level. Table S12. All-cause mortality, causes of anemia, without tacrolimus level. (DOCX 59 kb) [file 12882_2019_1244_MOESM1_ESM.docx]

**Supplementary 1 - Definitions**:

*Iron deficiency*

Iron deficiency anemia was defined as anemia (as defined by WHO) with any of the following:

- Ferritin <30 ng/ml or transferrin saturation <10% for eGFR≥60ml/min/1.73m2 (CKD-1-2).
- Ferritin <100 ng/ml or transferrin saturation <20% for eGFR<60ml/min/1.73m2 (CKD-3-5), according to KDOQI guidelines for non-hemodialysis CKD patients.
- Hypochromia (>10%) and microcytosis (MCV<80) in the absence of ferritin >100 ng/ml or transferrin saturation >20%.
- Border-line transferrin saturation (<15%) in case of hypochromia (>10%).

**Figure S1: Selection of patients**

1404 adult patients underwent a total of 1420 kidney transplantations during 2002-2016

- 265 patients (18.9%) excluded due to:

- Non-functioning graft after 180 days – 87 (6.2%)

- No follow-up – 86 (6.1%)

- Missing baseline data – 77 (5.5%)

- Additional organ transplantation – 15 (1.1%)

- 16 transplantations were excluded after achieving outcome during study period

1139 patients with functioning graft after 180 days

No anemia

727 patients

Anemia

412 patients

Table S1 - Composite outcome, with tacrolimus level

|  | | | HR | 95% C.I. | P value |
| --- | --- | --- | --- | --- | --- |
| Acute Rejection/AKI | | Early | 7.716 | 4.076-14.606 | .000 |
|  |  | Late | 1.720 | .755-3.915 | .196 |
| Infection | | Early | 3.380 | 1.627-7.022 | .001 |
|  |  | Late | .933 | .405-2.150 | .871 |
| Nutritional deficiencies | | Early | 2.571 | .758-8.722 | .130 |
|  |  | Late | .781 | .106-5.756 | .809 |
| Miscellaneous | | Early | 1.467 | .693-3.105 | .316 |
|  |  | Late | 1.068 | .546-2.087 | .848 |
| No reason found | | Early | .485 | .143-1.641 | .245 |
|  |  | Late | 1.026 | .538-1.954 | .939 |
| Mean age at transplant | | | 1.004 | .989-1.020 | .608 |
| Male sex | | | 1.294 | .910-1.841 | .151 |
| Mean duration of dialysis | | | 1.003 | .999-1.008 | .186 |
| Original renal disease | | |  |  |  |
|  | Glomerulonephritis | | .739 | .468-1.169 | .196 |
|  | CAKUT | | .689 | .335-1.418 | .312 |
|  | Diabetic nephropathy | | 1.187 | .608-2.318 | .615 |
|  | Polycystic kidney disease (PKD) | | .232 | .102-.531 | .001 |
|  | Genetic disease (excluding PKD) | | 1.071 | .341-3.364 | .906 |
|  | Chronic interstitial nephritis | | 1.639 | .721-3.725 | .238 |
| Diabetes | | | .836 | .437-1.597 | .587 |
| Cardiovascular | | | 1.673 | 1.137-2.461 | .009 |
| Living donor | | | .617 | .400-.951 | .029 |
| Mean donor age | | | 1.012 | .999-1.025 | .070 |
| HCV positive | | | 2.991 | 1.420-6.303 | .004 |
| Mean cold ischemia time (hours) | | | 1.000 | 1.000-1.000 | .192 |
| Induction therapy | | |  |  | .043 |
|  | ATG | | 2.390 | .990-5.770 | .053 |
|  | Interleukin-2 antagonists | | 2.255 | 1.011-5.028 | .047 |
|  | Other^a^ | | .928 | .297-2.895 | .897 |
| Immunosuppression (compared to tacrolimus) | | |  |  | .142 |
|  | Cyclosporine | | 1.795 | .975-3.303 | .060 |
|  | mTOR inhibitors | | 1.695 | .686-4.189 | .253 |
|  | Anti-metabolite & prednisone | | .625 | .176-2.220 | .467 |
| Length of hospitalization | | | 1.940 | 1.411-2.669 | .000 |
| eGFR | | | .987 | .977-.997 | .008 |
| Leukocytes | | | 1.051 | .992-1.114 | .094 |
| Mean level of tarcrolimus | | | 1.041 | .960-1.130 | .330 |

Abbreviations:

AKI, Acute kidney injury; CAKUT, Congenital anomalies of the kidney and urinary tract; HCV, Hepatitis C virus; ATG, Anti thymocyte globulin; mTOR, mammalian target of rapamycin; eGFR, Estimated glomerular filtration rate.

^a^Mostly Rituximab or IVIg

Table S2 - Death censored graft failure, with tacrolimus level

|  | | | HR | 95% C.I. | P value |
| --- | --- | --- | --- | --- | --- |
| Acute Rejection/AKI | | Early | 10.568 | 4.608-24.235 | <0.001 |
|  |  | Late | 3.084 | 1.155-8.233 | .025 |
| Infection | | Early | 2.819 | .881-9.018 | .081 |
|  |  | Late | .803 | .233-2.763 | .728 |
| Nutritional deficiencies | | Early | 1.291 | .165-10.130 | .808 |
|  |  | Late | 1.272 | .168-9.634 | 816 |
| Miscellaneous | | Early | 1.753 | .633-4.850 | .280 |
|  |  | Late | 1.233 | .528-2.881 | .628 |
| No reason found | | Early^a^ | ^a^ | ^a^ | ^a^ |
|  |  | Late | 1.253 | .516-3.039 | .619 |
| Mean age at transplant | | | .975 | .958-.992 | .004 |
| Male sex | | | .876 | .533-1.441 | .602 |
| Mean Donor age | | | 1.018 | .999-1.037 | .060 |
| Diabetes | | | .578 | .301-1.107 | .098 |
| Cardiovascular | | | 1.771 | .975-3.214 | .060 |
| Living donor | | | .450 | .253-.798 | .006 |
|  | ATG | | 2.374 | .766-7.356 | .134 |
|  | Interleukin-2 antagonists | | 1.480 | .518-4.230 | .464 |
|  | Other^b^ | | .983 | .209-4.636 | .983 |
| Immunosuppression (compared to tacrolimus) | | |  |  | .083 |
|  | Cyclosporine | | 2.503 | 1.140-5.494 | .022 |
|  | mTOR inhibitors | | 1.188 | .292-4.835 | .810 |
|  | Anti-metabolite & prednisone | | .320 | .040-2.562 | .283 |
| Length of hospitalization | | |  |  |  |
| eGFR | | | .989 | .976-1.002 | .090 |
| Mean level of tarcrolimus | | | 1.004 | .895-1.126 | .952 |

Abbreviations:

AKI, Acute kidney injury; ATG, Anti thymocyte globulin; mTOR, mammalian target of rapamycin; eGFR, Estimated glomerular filtration rate.

^a^No outcome events to report

^b^Mostly Rituximab or IVIg

Table S3 - All-cause mortality, with tacrolimus level

|  | | | HR | 95% C.I. | P value |
| --- | --- | --- | --- | --- | --- |
| Acute Rejection/AKI | | Early | 4.453 | 1.638-12.105 | .003 |
|  |  | Late | .508 | .111-2.326 | .383 |
| Infection | | Early | 4.216 | 1.650-10.772 | .003 |
|  |  | Late | 1.147 | .385-3.419 | .805 |
| Nutritional deficiencies | | Early | 4.237 | .916-19.599 | .065 |
|  |  | Late^a^ | ^a^ | ^a^ | ^a^ |
| Miscellaneous | | Early | 1.175 | .394-3.508 | .772 |
|  |  | Late | .733 | .243-2.216 | .582 |
| No reason found | | Early | .758 | .198-2.904 | .686 |
|  |  | Late | .686 | .268-1.757 | .432 |
| Mean age at transplant | | | 1.069 | 1.039-1.100 | .000 |
| Male sex | | | 1.702 | 1.010-2.869 | .046 |
| Mean donor age | | | 1.005 | .987-1.023 | .587 |
| Living donor | | | .401 | .219-.733 | .003 |
| Diabetes | | | 2.272 | 1.365-3.783 | .002 |
| Cardiovascular | | | 1.553 | .927-2.604 | .095 |
| HCV positive | | | 4.249 | 1.642-10.993 | .003 |
| Induction therapy | | |  |  | .370 |
|  | ATG | | 1.245 | .300-5.174 | .763 |
|  | Interleukin-2 antagonists | | 2.029 | .602-6.846 | .254 |
|  | Other^b^ | | 1.131 | .197-6.487 | .890 |
| Length of hospitalization | | | 3.025 | 1.940-4.717 | .000 |
| eGFR | | | .990 | .975-1.005 | .186 |
| Leukocytes | | | 1.080 | 1.000-1.166 | .049 |
| Mean level of tarcrolimus | | | 1.130 | 1.005-1.271 | .040 |

Abbreviations:

AKI, Acute kidney injury; HCV, Hepatitis C virus; ATG, Anti thymocyte globulin; eGFR, Estimated glomerular filtration rate.

^a^No outcome events to report

^b^Mostly Rituximab or IVIg

Table S4 - Composite outcome, presence of anemia, without tacrolimus level

|  | | HR | 95% C.I. | P value |
| --- | --- | --- | --- | --- |
| Mean age at transplant | | 1.006 | .994-1.018 | .336 |
| Male sex | | 1.254 | .944-1.667 | .118 |
| Mean duration of dialysis | | 1.002 | .999-1.005 | .136 |
| Original renal disease | |  |  | .001 |
|  | Glomerulonephritis | .837 | .589­1.188 | .319 |
|  | CAKUT | .748 | .425-1.315 | .313 |
|  | Diabetic nephropathy | 1.435 | .847-2.431 | .179 |
|  | Polycystic kidney disease (PKD) | .386 | .218-.683 | .001 |
|  | Genetic disease (excluding PKD) | .829 | .323-2.129 | .697 |
|  | Chronic interstitial nephritis | 2.329 | 1.143-4.744 | .020 |
| Diabetes | | .891 | .542-1.465 | .648 |
| Cardiovascular | | 1.347 | .984-1.845 | .063 |
| Living donor | | .531 | .372-.756 | .000 |
| Mean donor age | | 1.007 | .998-1.017 | .144 |
| HCV positive | | 1.913 | 1.170-3.128 | .010 |
| Donor CMV positive | | .910 | .666-1.244 | .555 |
| Induction therapy | |  |  | .023 |
|  | ATG | 1.517 | .968-2.377 | .069 |
|  | Interleukin-2 antagonists | 1.578 | 1.068-2.333 | .022 |
|  | Other^a^ | .810 | .420-1.561 | .529 |
| Immunosuppression (compared to tacrolimus) | |  |  | .005 |
|  | Cyclosporine | .491 | .235-1.023 | .057 |
|  | mTOR inhibitors | .753 | .324-1.750 | .510 |
|  | Anti-metabolite & prednisone | 1.072 | .422-2.727 | .883 |
| Length of hospitalization | | 1.919 | 1.449-2.542 | .000 |
| eGFR | | .986 | .978-.993 | .000 |
| Leukocytes | | 1.102 | 1.052-1.155 | .000 |
| Platelets | | .997 | .995-.999 | .001 |

Abbreviations:

CAKUT, Congenital anomalies of the kidney and urinary tract; HCV, Hepatitis C virus; ATG, Anti thymocyte globulin; mTOR, mammalian target of rapamycin; eGFR, Estimated glomerular filtration rate.

^a^Mostly Rituximab or IVIg

Table S5 - Composite outcome, severity of anemia, without tacrolimus level

|  | | HR | 95% C.I. | P value |
| --- | --- | --- | --- | --- |
| Mean age at transplant | | 11 | .999-1.023 | .074 |
| Male sex | | 1.035 | .777-1.378 | .816 |
| Mean duration of dialysis | | 1.003 | 1.000-1.006 | .054 |
| Original renal disease | |  |  | .001 |
|  | Glomerulonephritis | .842 | .593-1.194 | .334 |
|  | CAKUT | .773 | .444-1.348 | .365 |
|  | Diabetic nephropathy | 1.295 | .761-2.206 | .340 |
|  | Polycystic kidney disease (PKD) | .370 | .207-.661 | .001 |
|  | Genetic disease (excluding PKD) | 1.190 | .467-3.033 | .715 |
|  | Chronic interstitial nephritis | 2.516 | 1.235-5.126 | .011 |
| Diabetes | | .970 | .592-1.591 | .905 |
| Cardiovascular | | 1.369 | 1.003-1.869 | .048 |
| Living donor | | .621 | .435-.885 | .008 |
| Mean donor age | | 1.008 | .998-1.018 | .099 |
| HCV positive | | 1.963 | 1.200-3.213 | .007 |
| Donor CMV positive | | .899 | .656-1.231 | .507 |
| Induction therapy | |  |  | .022 |
|  | ATG | 1.447 | .927-2.260 | .104 |
|  | Interleukin-2 antagonists | 1.657 | 1.126-2.439 | .010 |
|  | Other^a^ | .895 | .467-1.713 | .737 |
| Immunosuppression (compared to tacrolimus) | |  |  | .034 |
|  | Cyclosporine | 1.416 | .918-2.186 | .116 |
|  | mTOR inhibitors | 1.944 | 1.080-3.500 | .027 |
|  | Anti-metabolite & prednisone | 1.769 | .834-3.755 | 137 |
| Length of hospitalization | | 1.881 | 1.429-2.476 | .000 |
| eGFR | | .988 | .980-.995 | .002 |

Abbreviations:

CAKUT, Congenital anomalies of the kidney and urinary tract; HCV, Hepatitis C virus; CMV, Cytomegalovirus; ATG, Anti thymocyte globulin; mTOR, mammalian target of rapamycin; eGFR, Estimated glomerular filtration rate.

^a^Mostly Rituximab or IVIg

Table S6 - Composite outcome, causes of anemia, without tacrolimus level

|  | | HR | 95% C.I. | P value |
| --- | --- | --- | --- | --- |
| Mean age at transplant | | 1.009 | .997-1.022 | .136 |
| Male sex | | 1.174 | .887-1.554 | .261 |
| Mean duration of dialysis | | 1.002 | .999-1.005 | .221 |
| Original renal disease | |  |  | .004 |
|  | Glomerulonephritis | .840 | .591-1.195 | .333 |
|  | CAKUT | .720 | .410-1.266 | .254 |
|  | Diabetic nephropathy | 1.148 | .673-1.958 | .613 |
|  | Polycystic kidney disease (PKD) | .369 | .207-.658 | .001 |
|  | Genetic disease (excluding PKD) | .854 | .332-2.199 | .744 |
|  | Chronic interstitial nephritis | 1.994 | .968-4.106 | .061 |
| Diabetes | | 1.003 | .607-1.657 | .991 |
| Cardiovascular | | 1.348 | .983-1.848 | .063 |
| Living donor | | .528 | .372-.749 | .000 |
| Mean donor age | | 1.011 | 1.001-1.021 | .028 |
| HCV positive | | 1.899 | 1.165-3.096 | .010 |
| Induction therapy | |  |  | .029 |
|  | ATG | 1.542 | .987-2.407 | .057 |
|  | Interleukin-2 antagonists | 1.590 | 1.079-2.342 | .019 |
|  | Other^a^ | .875 | .456-1.679 | .688 |
| Immunosuppression (compared to tacrolimus) | |  |  | .062 |
|  | Cyclosporine | 1.541 | .996-2.382 | .052 |
|  | mTOR inhibitors | 1.707 | .947-3.077 | .075 |
|  | Anti-metabolite & prednisone | 1.493 | .699-3.191 | .301 |
| Length of hospitalization | | 1.968 | 1.487-2.604 | .000 |
| eGFR | | .987 | .979-.995 | .001 |
| Leukocytes | | 1.070 | 1.023-1.120 | .003 |

Abbreviations:

CAKUT, Congenital anomalies of the kidney and urinary tract; HCV, Hepatitis C virus; ATG, Anti thymocyte globulin; mTOR, mammalian target of rapamycin; eGFR, Estimated glomerular filtration rate.

^a^Mostly Rituximab or IVIg

Table S7 - Death censored graft failure, presence of anemia, without tacrolimus level

|  | | HR | 95% C.I. | P value |
| --- | --- | --- | --- | --- |
| Mean age at transplant | | .970 | .955-.986 | .986 |
| Male sex | | .823 | .545-1.245 | .357 |
| Original renal disease | |  |  | .136 |
|  | Glomerulonephritis | .765 | .483-1.210 | .252 |
|  | CAKUT | .541 | .270-1.087 | .084 |
|  | Diabetic nephropathy | .578 | .227-1.471 | .250 |
|  | Polycystic kidney disease (PKD) | .357 | .150-.851 | .020 |
|  | Genetic disease (excluding PKD) | .830 | .281-2.456 | .737 |
|  | Chronic interstitial nephritis | 1.522 | .531-4.357 | .434 |
| Diabetes | | .784 | .354-1.733 | .547 |
| Cardiovascular | | 1.522 | .912­-2.538 | .108 |
| Living donor | | .498 | .302-.823 | .007 |
| Mean donor age | | 1.010 | .995­-1.024 | .185 |
| HCV positive | | 1.705 | .827-3.517 | .148 |
| Donor CMV positive | | .873 | .539-1.413 | .580 |
| Induction therapy | |  |  | .083 |
|  | ATG | 1.958 | 1.068-3.590 | .030 |
|  | Interleukin-2 antagonists | 1.816 | .795­-4.146 | .157 |
|  | Other^a^ | .512 | .069-3.777 | .512 |
| Immunosuppression (compared to tacrolimus) | |  |  | .140 |
|  | Cyclosporine | 1.714 | .908-3.235 | .097 |
|  | mTOR inhibitors | 1.258 | .707-2.242 | .435 |
|  | Anti-metabolite & prednisone | .760 | .307-1.880 | .553 |
| Length of hospitalization | | 1.380 | .931-2.046 | .109 |
| eGFR | | .980 | .970-.991 | .000 |
| Leukocytes | | 1.001 | .937-1.070 | .967 |

Abbreviations:

CAKUT, Congenital anomalies of the kidney and urinary tract; HCV, Hepatitis C virus; CMV, Cytomegalovirus; ATG, Anti thymocyte globulin; mTOR, mammalian target of rapamycin; eGFR, Estimated glomerular filtration rate.

^a^Mostly Rituximab or IVIg

Table S8 - Death censored graft failure, Severity of anemia, without tacrolimus level

|  | | HR | 95% C.I. | P value |
| --- | --- | --- | --- | --- |
| Mean age at transplant | | .973 | .957-.988 | .001 |
| Male sex | | .684 | .444-1.056 | .086 |
| Mean duration of dialysis | | 1.002 | .998-1.006 | .313 |
| Original renal disease | |  |  | .106 |
|  | Glomerulonephritis | .720 | .454-1.141 | .162 |
|  | CAKUT | .546 | .275-1.085 | .084 |
|  | Diabetic nephropathy | .536 | .210-1.370 | .193 |
|  | Polycystic kidney disease (PKD) | .352 | .147-.844 | .019 |
|  | Genetic disease (excluding PKD) | .885 | .300-2.612 | .825 |
|  | Chronic interstitial nephritis | 1.574 | .546-4.541 | .401 |
| Diabetes | | .835 | .379-1.839 | .654 |
| Cardiovascular | | 1.401 | .839-2.341 | .198 |
| Living donor | | .568 | .337-.957 | .034 |
| HCV positive | | 1.625 | .784-3.370 | .192 |
| Donor CMV positive | | .955 | .581-1.571 | .857 |
| Induction therapy | |  |  | .152 |
|  | ATG | 1.723 | .919-3.231 | .090 |
|  | Interleukin-2 antagonists | 1.343 | .759-2.377 | .312 |
|  | Other^a^ | .783 | .316-1.940 | .598 |
| Immunosuppression (compared to tacrolimus) | |  |  | .151 |
|  | Cyclosporine | 1.790 | .967-3.312 | .064 |
|  | mTOR inhibitors | 1.595 | .692-3.679 | .273 |
|  | Anti-metabolite & prednisone | .394 | .052-2.974 | .366 |
| Length of hospitalization | | 1.370 | .920-2.038 | .121 |
| eGFR | | .982 | .971-.992 | .001 |
| Mean donor age | | 1.009 | .995-1.023 | .222 |

Abbreviations:

CAKUT, Congenital anomalies of the kidney and urinary tract; HCV, Hepatitis C virus; CMV, Cytomegalovirus; ATG, Anti thymocyte globulin; mTOR, mammalian target of rapamycin; eGFR, Estimated glomerular filtration rate.

^a^Mostly Rituximab or IVIg

Table S9 - Death censored graft failure, causes of anemia, without tacrolimus level

|  | | HR | 95% C.I. | P value |
| --- | --- | --- | --- | --- |
| Mean age at transplant | | .971 | .955-.987 | .001 |
| Male sex | | .886 | .584-1.344 | .568 |
| Mean duration of dialysis | | 1.002 | .997-1.006 | .477 |
| Original renal disease | |  |  | .070 |
|  | Glomerulonephritis | .733 | .461-1.167 | .191 |
|  | CAKUT | .462 | .227-.940 | .033 |
|  | Diabetic nephropathy | .431 | .167-1.111 | .082 |
|  | Polycystic kidney disease (PKD) | .358 | .149-.856 | .021 |
|  | Genetic disease (excluding PKD) | .685 | .229-2.044 | .497 |
|  | Chronic interstitial nephritis | 1.270 | .434-3.713 | .663 |
| Diabetes | | .921 | .414-2.045 | .839 |
| Cardiovascular | | 1.612 | .970-2.679 | .066 |
| Living donor | | .460 | .274-.771 | .003 |
| Mean donor age | | 1.013 | .998-1.028 | .083 |
| HCV positive | | 1.712 | .832-3.524 | .144 |
| Induction therapy | |  |  | .121 |
|  | ATG | 1.816 | .966-3.412 | .064 |
|  | Interleukin-2 antagonists | 1.284 | .725-2.273 | .391 |
|  | Other^a^ | .818 | .327-2.042 | .666 |
| Immunosuppression (compared to tacrolimus) | |  |  | .047 |
|  | Cyclosporine | 2.071 | 1.122-3.822 | .020 |
|  | mTOR inhibitors | 1.446 | .611-3.424 | .401 |
|  | Anti-metabolite & prednisone | .227 | .029-1.762 | .156 |
| eGFR | | .982 | .971-.992 | .001 |
| Leukocytes | | 1.007 | .940-1.078 | .852 |

Abbreviations:

CAKUT, Congenital anomalies of the kidney and urinary tract; HCV, Hepatitis C virus; ATG, Anti thymocyte globulin; mTOR, mammalian target of rapamycin; eGFR, Estimated glomerular filtration rate.

^a^Mostly Rituximab or IVIg

Table S10 - All-cause mortality, presence of anemia, without tacrolimus level

|  | | HR | 95% C.I. | P value |
| --- | --- | --- | --- | --- |
| Mean age at transplant | | 1.083 | 1.058-1.109 | .000 |
| Male sex | | 1.641 | 1.103-2.442 | .015 |
| Mean duration of dialysis | | 1.006 | 1.001­-1.010 | .025 |
| Original renal disease | |  |  | .001 |
|  | Glomerulonephritis | 1.013 | .572-1.794 | .965 |
|  | CAKUT | .815 | .276-2.405 | .711 |
|  | Diabetic nephropathy | 2.081 | 1.040-4.163 | .038 |
|  | Polycystic kidney disease (PKD) | .316 | .137-.731 | .007 |
|  | Genetic disease (excluding PKD) | .899 | .120-6.717 | .917 |
|  | Chronic interstitial nephritis | 4.339 | 1.607-11.713 | .004 |
| Diabetes | | 1.077 | .555-2.093 | .826 |
| Cardiovascular | | 1.508 | .986-2.305 | .058 |
| Living donor | | .503 | .300-.841 | .009 |
| Mean donor age | | .998 | .985-1.012 | .819 |
| HCV positive | | 2.647 | 1.340-5.229 | .005 |
| Donor CMV positive | | .897 | .585-1.375 | .617 |
| Previous renal transplantation | | .818 | .381-1.755 | .606 |
| Induction therapy | |  |  | .027 |
|  | ATG | 1.158 | .581-2.309 | .676 |
|  | Interleukin-2 antagonists | 2.016 | 1.157­-3.512 | .013 |
|  | Other^a^ | 1.112 | .421-2.935 | .831 |
| Immunosuppression (compared to tacrolimus) | |  |  | .000 |
|  | Cyclosporine | 1.092 | .570­-2.093 | .791 |
|  | mTOR inhibitors | 3.374 | 1.389-8.194 | .007 |
|  | Anti-metabolite & prednisone | 4.448 | 1.905­-10.385 | .001 |
| Length of hospitalization | | 2.834 | 1.888-4.256 | .000 |
| eGFR | | .995 | .984-1.006 | .370 |

Abbreviations:

CAKUT, Congenital anomalies of the kidney and urinary tract; HCV, Hepatitis C virus; CMV, Cytomegalovirus; ATG, Anti thymocyte globulin; mTOR, mammalian target of rapamycin; eGFR, Estimated glomerular filtration rate.

^a^Mostly Rituximab or IVIg

Table S11 - All-cause mortality, severity of anemia, without tacrolimus level

|  | | HR | 95% C.I. | P value |
| --- | --- | --- | --- | --- |
| Mean age at transplant | | 1.078 | 1.053-1.103 | .000 |
| Male sex | | 1.649 | 1.094-2.485 | .017 |
| Mean duration of dialysis | | 1.004 | 1.000-1.009 | .054 |
| Original renal disease | |  |  | .001 |
|  | Glomerulonephritis | .956 | .542-1.685 | .876 |
|  | CAKUT | .691 | .233-2.048 | .505 |
|  | Diabetic nephropathy | 2.282 | 1.134­-4.591 | .021 |
|  | Polycystic kidney disease (PKD) | .316 | .137-.730 | .007 |
|  | Genetic disease (excluding PKD) | .684 | .090-5.192 | .713 |
|  | Chronic interstitial nephritis | 4.239 | 1.569-11.456 | .004 |
| Diabetes | | .912 | .463-1.797 | .790 |
| Cardiovascular | | 1.426 | .933-2.179 | .102 |
| Living donor | | .462 | .277-.771 | .003 |
| Mean donor age | | 1.003 | .989-1.017 | .668 |
| HCV positive | | 2.517 | 1.276-4.963 | .008 |
| Donor CMV positive | | 1.032 | .672-1.584 | .886 |
| Induction therapy | |  |  | .014 |
|  | ATG | 1.470 | .742-2.910 | .269 |
|  | Interleukin-2 antagonists | 2.303 | 1.313-4.040 | .004 |
|  | Other^a^ | 1.124 | .427-2.957 | .813 |
| Immunosuppression (compared to tacrolimus) | |  |  | .001 |
|  | Cyclosporine | 1.005 | .525-1.926 | .988 |
|  | mTOR inhibitors | 3.302 | 1.373-7.939 | .008 |
|  | Anti-metabolite & prednisone | 3.811 | 1.625-8.938 | .002 |
| Length of hospitalization | | 2.594 | 1.714-3.927 | .000 |
| eGFR | | .996 | .984-1.007 | .456 |
| Leukocytes | | 1.129 | 1.063-1.199 | .000 |
| Platelets | | .997 | .994-1.000 | .032 |

Abbreviations:

CAKUT, Congenital anomalies of the kidney and urinary tract; HCV, Hepatitis C virus; CMV, Cytomegalovirus; ATG, Anti thymocyte globulin; mTOR, mammalian target of rapamycin; eGFR, Estimated glomerular filtration rate.

^a^Mostly Rituximab or IVIg

Table S12 - All-cause mortality, causes of anemia, without tacrolimus level

|  | | HR | 95% C.I. | P value |
| --- | --- | --- | --- | --- |
| Mean age at transplant | | 1.068 | 1.046-1.091 | .000 |
| Male sex | | 1.654 | 1.114-2.455 | .013 |
| Diabetes | | 2.102 | 1.427-3.095 | .000 |
| Cardiovascular | | 1.378 | .900-2.111 | .140 |
| Living donor | | .372 | .229-.605 | .000 |
| Mean donor age | | 1.007 | .994-1.021 | .298 |
| HCV positive | | 2.583 | 1.328-5.023 | .005 |
| Induction therapy | |  |  | .030 |
|  | ATG | 1.229 | .628-2.407 | .547 |
|  | Interleukin-2 antagonists | 1.959 | 1.131-3.393 | .016 |
|  | Other^a^ | .865 | .332-2.254 | .766 |
| Immunosuppression (compared to tacrolimus) | |  |  | .004 |
|  | Cyclosporine | 1.132 | .590-2.171 | .709 |
|  | mTOR inhibitors | 1.923 | .813-4.546 | .136 |
|  | Anti-metabolite & prednisone | 4.236 | 1.827-9.821 | .001 |
| Length of hospitalization | | 2.845 | 1.909-4.240 | .000 |
| eGFR | | .994 | .983-1.006 | .312 |
| Leukocytes | | 1.097 | 1.034-1.163 | .002 |

Abbreviations:

HCV, Hepatitis C virus; ATG, Anti thymocyte globulin; mTOR, mammalian target of rapamycin; eGFR, Estimated glomerular filtration rate.

^a^Mostly Rituximab or IVIg
